# The Effect of Saffron (Crocus sativus L.) Supplementation on Renal Function: A Systematic Review and Meta-Analysis of Randomized Controlled Clinical Trials

**DOI:** 10.1155/2022/9622546

**Published:** 2022-08-29

**Authors:** Elham Karimi, Farnaz Shahdadian, Amir Hadi, Mohammad-Aref Tarrahi, Mohammad Javad Tarrahi

**Affiliations:** ^1^Research Development Center, Arash Women's Hospital, Tehran University of Medical Sciences, Tehran, Iran; ^2^Department of Clinical Nutrition, School of Nutrition and Food Science, Food Security Research Center, Isfahan University of Medical Sciences, Isfahan, Iran; ^3^Halal Research Center of IRI, Food and Drug Administration, Ministry of Health and Medical Education, Tehran, Iran; ^4^Department of Community Nutrition, School of Nutrition and Food Science, Isfahan University of Medical Sciences, Isfahan, Iran; ^5^Department of Epidemiology and Biostatistics, School of Health, Isfahan University of Medical Sciences, Isfahan, Iran

## Abstract

**Background:**

The present systematic review and meta‐analysis of randomized controlled trials (RCTs) aimed at determining the effect of saffron supplementation on renal function.

**Methods:**

Electronic databases were searched up to February 2021. The risk of bias in individual studies was assessed using the Cochrane Collaboration tool. The overall weighted mean difference (WMD) and their 95% confidence intervals (CIs) were calculated using random-effect models. *P* < 0.05 was considered statistically significant.

**Results:**

A total of 11 trials were included in this study. Saffron had beneficial effect on BUN (WMD = −0.69 mg/dl; 95% CI, −1.36 to −0.01; *P*=0.046) compared to placebo, with significant heterogeneity (*I*^2^ = 49.6%, *P*=0.037). However, it had no significant effect on serum Cr (WMD = 0.04 mg/dl; 95% CI: −0.01 to 0.09; *P*=0.127).

**Conclusion:**

It seems that saffron supplementation had no significant effect on Cr as a renal function factor. However, BUN reduction was significant in the saffron group compared to placebo.

## 1. Introduction

Saffron (*Crocus sativus* L.) is a bulbous perennial of the Iridaceae family that is widely cultivated in Iran [1]. Saffron is used as a valuable spice for flavoring and coloring, as well as in traditional herbal medicine [2, 3]. The possible beneficial effects of saffron are related to a number of its components including crocin, picrocrocin, safranal, and crocetin [4, 5]. Also, it is a carotenoid- and flavonoid-rich spice that has been studied due to its pharmacological activities such as antioxidants [6], nerve relaxants [7], anti-inflammatory [8], anticonvulsant [9], and antitumor [10] effects. Moreover, it is effective in treating some conditions such as hypertension (HTN), dyslipidemia, type 2 diabetes mellitus (T2DM), and liver disease [4, 11, 12].

Oxidative stress, systematic inflammation, and complications of chronic diseases are related to the pathogenesis of renal disorders [13, 14]. Evidence showed an increasing prevalence of renal dysfunction following the development of uncommunicable diseases such as T2DM, HTN, cardiovascular disease (CVD), and obesity [15]. Also, 4.6% of total mortality is related to chronic kidney disease (CKD) and CVD deaths that resulted from impaired kidney function [16]. So, the management of underlying conditions has an important role in the prevention and control of CKD. Herbal medicines, as one type of dietary supplement, have been proposed to have beneficial roles in the improvement of renal function and the causes of kidney disorders [17–21].

Several studies have investigated the effect of saffron or its components on Cr and BUN as markers of renal function. Some of them did not show any significant effect of saffron on Cr and BUN [22–24], while others reported a significant reduction [25]. On the other hand, another study indicated an increase in Cr and BUN with saffron consumption [1]. Due to the discrepancy in the findings of the current literature, we conducted this study to evaluate the effects of saffron supplementation on renal function tests including Cr and BUN among the adult population using a systematic review and meta-analysis of randomized controlled trials.

## 2. Methods

On the basis of the Preferred Reporting Items for Systematic Reviews and Meta‐Analysis (PRISMA), this study was conducted and reported [26].

### 2.1. Search Strategy

tWe searched ISI Web of Science (http://www.webofscience.com), Scopus (http://www.scopus.com), and PubMed (http://www.ncbi.nlm.nih.gov/pubmed) databases from the earliest available studies to March 2021, via the following keywords: (“picrocrocin” OR “crocetin” OR “safranal” OR “saffron” OR “Crocus sativus” OR “crocin”) AND (“Renal function” OR “Renal function test” OR “Kidney function” OR “Kidney function test” OR “Blood urea nitrogen” OR “BUN” OR “Urea” OR “Creatinine”). The search strategy of the selected databases is shown in Table 1. References of eligible studies and related reviews were also scrutinized for additional articles. Duplicate publications were removed after exporting all searched articles to EndNote software (version X8.1, for Windows, Thomson Reuters, Philadelphia, PA, USA), to simplify the study selection process.

### 2.2. Study Selection

Once all of the search results were exported, irrelevant articles were excluded by screening titles and abstracts by two investigators (E. K and A. H). All included studies were identified by retrieving the full text of the remaining articles. Inclusion criteria were as follows: (1) being an RCT with crossover or parallel design and (2) exploring the effect of saffron consumption on renal function tests including BUN, Urea, or Cr. Exclusion criteria were as follows: studies that included subjects aged <18 years, who are pregnant, or who are lactating women; and studies with noncomparative data, duration of intervention ≤2 weeks, and lack of outcome measures.

### 2.3. Data Extraction

Two independent authors (E.K and F.S) did data extraction. The following data were extracted: [1] participants' information (health status, mean age, gender, and body mass index [BMI]); [2] study characteristics (first author's name, date of publication, study location, sample size, and study design); [3] intervention details (form and dose of saffron, duration of follow-up, and intervention of the control group); and [4] mean ± standard deviation (SD) (or mean ± standard error (SE)) of change in serum BUN and Cr in each group of intervention and control. For studies with missing data, the authors were sent emails requesting details of these data.

### 2.4. Quality Assessment

The quality of the studies was evaluated independently by 2 reviewers (E. K and MA. T) using the Cochrane Collaboration tool for the systematic reviews of interventions [27]. The following methodological domains were considered random sequence generation, allocation concealment, blinding, incomplete outcome data, selective reporting, and other bias. Each item was scored as a low, unclear, or high risk of bias.

### 2.5. Grading of the Evidence

The certainty of the evidence was assessed via the GRADE tool [28]. On the basis of this approach, there are five categories of evidence in terms of quality ranging from high to very low. Evidence was graded based on publication bias (small-study effects significantly evident), study restrictions (weight of datasets revealing the risk of bias based on the Cochrane risk of bias tool), imprecision (the 95% confidence intervals (95% CIs) for mean difference and risk estimates are wide or cross a minimally important difference) inconsistency (meaningful unjustifiable interstudy heterogeneity, I^2^ ≥ 50% and *P* < 0.10), and indirectness (existence of factors that diminish the generalizability of the results).

### 2.6. Statistical Analysis

The meta-analysis was conducted by using the STATA software (version 11.0; Stata Corporation). To estimate the pooled effect, all of the related data were collected in mean ± SD for Cr and BUN in a similar unit. Moreover, the Follmann method was implemented to compute SD for the net changes [29]. In studies where the SE was reported, SD was calculated as follows: SD = SE × sqrt (*n*) (*n*: number of participants in each group). Weighted mean differences (WMD) and 95% confidence intervals were calculated for BUN and Cr by using a random-effect model. Heterogeneity between studies was examined using the I-squared (*I*^2^) index and tau squares. If the *I*^2^ was >50%, heterogeneity existed between the included trials. Also, Egger's regression intercept, Begg's test, and funnel plot were run to assess the presence of publication bias. In this analysis, *P* < 0.05 was considered statistically significant.

## 3. Results

### 3.1. Search Results

**Figure 1:**
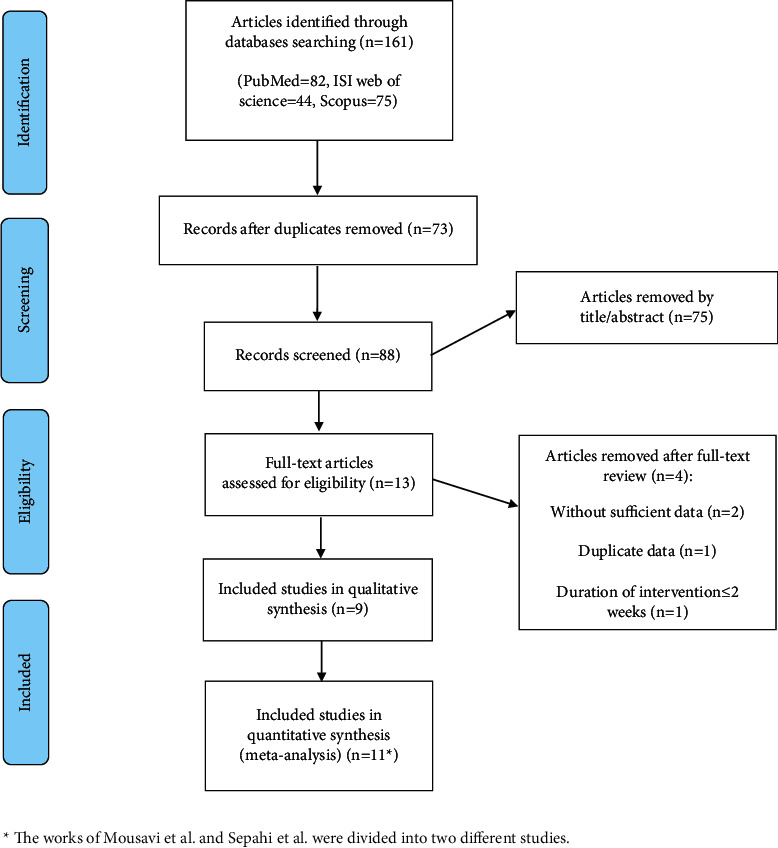
PRISMA flow diagram of the study selection process.

A total of 161 articles were identified in the initial search. After removing duplicates, 88 articles were reviewed based on the title and abstract and 75 unrelated studies were excluded at this stage. The remaining articles [11] were resumed and reviewed based on the full text. Two out of nine enrolled articles were divided into two different studies [23, 30]. Finally, 11 trials were included in this systematic review and meta-analysis. The process of study identification is summarized in the PRISMA flow diagram (Figure 1).

### 3.2. Trials Characteristics

A total of 577 participants from 11 trials were included in this systematic review and meta-analysis. These studies were published between 2011 and 2019. The design of all the enrolled studies was parallel, and they were carried out in Iran. Participants' ages ranged from 21.8 to 56.63 years, and the mean body mass index (BMI) ranged from 21.5 to 29.9. The duration of the intervention varied from 4 to 12 weeks. Kianbakht and Mousavi studies [30, 31] have recruited only males and the others both gender [22–25, 32–34]. The dosage of saffron was between 15 and 100 mg/day, and crocin ranged from 5 to 30 mg/day. Saffron and crocin were used in six [22, 24, 25, 31–33] and two [23, 34] studies, respectively, and only one study [30] used both of them. Participants were selected from individuals with schizophrenia [30], major depressive disorder (MDD) [24], diabetes mellitus (DM) [22, 23, 25, 32, 33], and healthy populations [31, 34]. Based on the Cochrane bias assessment tool, four studies ranked as good [22, 24, 25, 33], one as fair [23], and four as poor qualities [30–32, 34]. The main characteristics of eligible trials are present in Table 2, and the results of the risk of bias assessment of included studies in Table 3.

### 3.3. Findings from Meta-Analysis

#### 3.3.1. Saffron and Creatinine

**Figure 2:**
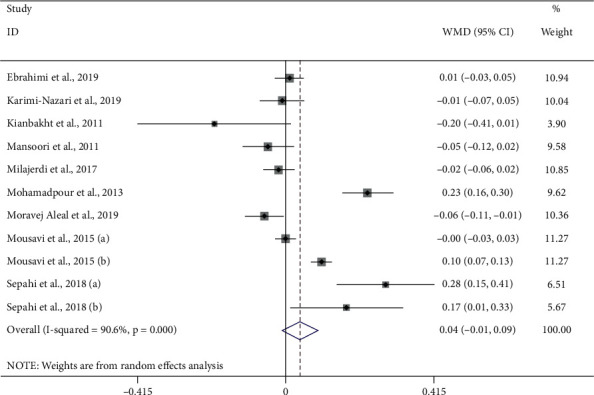
Forest plot of the effect of saffron supplementation on creatinine.

**Figure 3:**
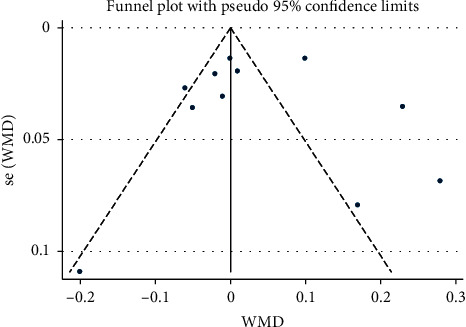
Funnel plot of the effect of saffron supplementation on creatinine.

The effect of saffron consumption on creatinine was examined in 11 studies [22–25, 30–33]. Overall, meta-analysis revealed that saffron had no beneficial effect on Cr (WMD = 0.04 mg/dl; 95% CI: −0.01 to 0.09; *P*=0.127) (Figure 2). Significant heterogeneity was observed among the effect size of the included studies (*I*^2^ = 90.6%, *P* < 0.001). Publication bias was not observed among the included studies (*P*=0.867, Egger's test, and *P*=0.639, Begg's test) (Figure 3).

#### 3.3.2. Saffron and BUN

**Figure 4:**
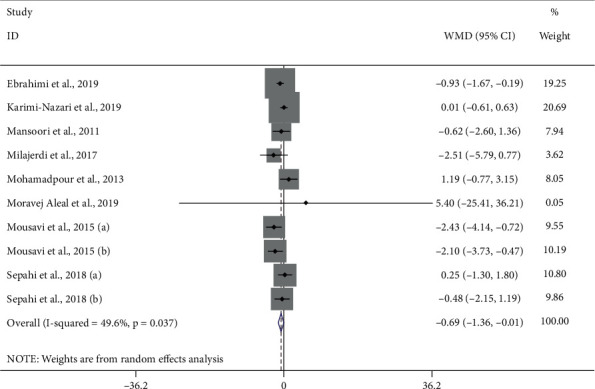
Forest plot of the effect of saffron supplementation on BUN.

**Figure 5:**
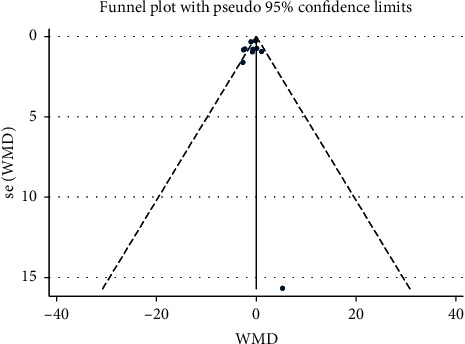
Funnel plot of the effect of saffron supplementation on BUN.

Ten studies [22–25, 30, 32–34] reported the effect of saffron supplementation on BUN. Saffron had beneficial effect on BUN (WMD = −0.69 mg/dl; 95% CI, −1.36 to −0.01; *P*=0.046) compared to placebo (Figure 4), with significant heterogeneity (*I*^2^ = 49.6%, *P*=0.037). Furthermore, there was no evidence of publication bias (*P*=0.543, Egger's test, and *P*=0.655, Begg's test) (Figure 5).

#### 3.3.3. The Results of the GRADE Assessment of the Certainty of the Evidence

The GRADE assessment of the certainty of the evidence is shown in Table 4. The evidence was rated as very low for the effects of saffron consumption on Cr and low for BUN.

## 4. Discussion

This study is the first comprehensive systematic review and meta-analysis of randomized controlled clinical trials to clarify the effect of saffron supplementation on renal function. Although our findings suggested that saffron has a beneficial effect on BUN, any significant effect was not observed on Cr levels in the saffron group compared to placebo.

The results of previous studies on the effect of saffron supplementation on the metabolic parameters were inconsistent. Several previous meta-analyses observed that saffron administration could play an important role in the improvement of metabolic indices. A systematic review and meta-analysis on the effect of saffron on blood glucose and lipid profile suggested that saffron has a beneficial role in the improvement of serum concentration of total cholesterol, triglycerides (TG), and high-density lipoprotein cholesterol (HDL-C). However, a significant effect was not observed on fasting plasma glucose and low-density lipoprotein cholesterol (LDL-C) [35]. Besides, the other study showed that saffron had a favorable effect on body weight, waist circumference, and diastolic blood pressure, and conversely, the improvement in lipid profile, fasting plasma glucose (FPG), fasting insulin, hemoglobin A1c (HbA1c), and BMI was not observed by saffron [36]. Several meta-analyses showed that saffron administration had no significant effect on inflammatory cytokines and homeostatic model assessment for insulin resistance (HOMA-IR) [4, 37]. However, animal studies suggested that saffron might have a role in BUN and Cr reduction in diabetic rats [38]. Another animal study showed that crocin declined the increased plasma levels of BUN and Cr in rats with DM that this effect resulted from its antioxidant properties [39]. This inconsistent result might refer to administrated saffron dosage in animal studies that was higher than that prescribed in human studies.

Several points should be accountable in the explanation of these null results. Most of the included articles recruited nonkidney disease individuals who were not on the upper end of abnormal cut-points for BUN and Cr, which might lessen the chance of achieving significant changes in these features following the intervention. Except for four studies, other studies were conducted among subjects with different diseases and medication use including antidepressant, antipsychotics, statins, and oral hypoglycemic agents. These drugs might cause an elevation in BUN and Cr, which diminished the possible efficacy of saffron on renal function tests. Besides, the sample size of most of the studies was small for assessing the effect of saffron on renal function. Also, dosage and duration of supplementation were different between studies might influence the results and the quality of half of the studies was poor to assess the effect of saffron on renal function. Furthermore, this point should be considered that there were various types of saffron that have different amounts of components and constituents that might change its efficacy [40]. Lastly, significant heterogeneity of the included studies should be considered while interpreting the findings. The observed heterogeneity may be contributed to differences in sample size, target population health status and age, the protocol of saffron intervention in terms of dose and duration, and source of saffron.

Previous studies demonstrated that the progression of kidney damage is related to the production of free radicals, oxidative stress, and systematic inflammation [13, 14, 41]. Saffron as an antioxidant agent might attenuate the inflammation levels in the body and protect the kidney from damage. Saffron contains carotenoids and flavonoids that are involved in the free radical scavenging activity of saffron [42]. Another mechanism for the reduction of oxidative stress by saffron is related to the ability of saffron to decrease the serum nitric oxide (NO) and malondialdehyde (MDA) and increase glutathione-S-transferase activity (GST), which increase the antioxidant capacity to remove free radicals [43]. On the other hand, the blood-glucose-lowering effect of saffron plays a role in the prevention of diabetic nephropathy progression [44, 45]. The mechanisms contributed to the glucose-lowering activity of saffron are including prevention of reabsorption of renal glucose, increase in insulin production via *β*-cells regeneration, and improvement of glucose uptake via pathways that are mediated by adenosine monophosphate-activated protein kinase/acetyl-CoA carboxylase (AMPK/ACC) and mitogen-activated protein kinases (MAPKs) [12, 46].

Although saffron is known as a safe herbal medicine with low toxicity on the normal cells of the body, a daily dosage of up to 1.5 g/day was considered safe in human studies [47, 48]. The toxic effect appeared in dosages above 5 g/day and near 20 g/daily dosage of saffron known as a lethal dosage [47]. Most of the included studies did not show any significant side effects following saffron consumption, except in one study [23]. The reported side effect of saffron supplementation in Sepahi et al. study included foot swelling, stomach ache, increased appetite, burning of the eyes, redness, swelling of the eyes, and subconjunctival hemorrhage, which were seen in both placebo and crocin treatment groups, but there were no significant differences between the two groups in case of mentioned side effects [23]. Moreover, the study that evaluated the safety of saffron supplementation in healthy subjects showed that saffron elevated the BUN and Cr, but was not clinically significant [1].

Although the current study is among the first that comprehensively reviewed the current literature regarding the role of saffron on renal function, some points should be taken into account as limitations. First, since all studies were performed among the Iranian population, the generalizability of findings is diminished. Second, the sample size of most of the studies that have evaluated the effectiveness of saffron in the improvement of renal function indices was small. Third, varying levels of the quality of the evidence and the highly statistically signiﬁcant heterogeneity among studies due to different characteristics of the population, aspects of methodology, and disparity in the intervention may have masked significant results on the effect of saffron. In addition, the protocol of the current study was not pre-registered in the PROSPERO database.

## 5. Conclusion

In conclusion, this comprehensive systematic review and meta-analysis of randomized controlled clinical trials showed no significant effect of saffron on Cr as the factor for assessment of renal function. However, BUN reduction was signiﬁcant in the saffron group compared to placebo. Further clinical trials with larger sample size, longer duration, and higher doses of saffron should be conducted exclusively among patients with renal dysfunction to clarify the beneficial role of saffron consumption on kidney function.

## Figures and Tables

**Table 1:** Search strategy of the selected databases.

PubMed
Search hits: 82
(“picrocrocin” [Supplementary Concept] OR “picrocrocin” [All Fields] OR (“trans sodium crocetinate” [Supplementary Concept] OR “trans sodium crocetinate” [All Fields] OR “crocetin” [All Fields]) OR (“safranal” [Supplementary Concept] OR “safranal” [All Fields]) OR (“crocus” [MeSH Terms] OR “crocus” [All Fields] OR “saffron” [All Fields]) OR “Crocus sativus” [All Fields] OR (“crocin” [Supplementary Concept] OR “crocin” [All Fields] OR “crocin s” [All Fields] OR “crocins” [All Fields])) AND (“Renal function” [All Fields] OR “Renal function test”[All Fields] OR “Kidney function”[All Fields] OR “Kidney function test”[All Fields] OR “Blood urea nitrogen”[All Fields] OR (“creatinin” [All Fields] OR “creatinine” [MeSH Terms] OR “creatinine” [All Fields] OR “creatinines” [All Fields]) OR “BUN” [All Fields] OR (“urea” [MeSH Terms] OR “urea” [All Fields]))
Scopus
Search hits: 121
((TITLE-ABS-KEY (picrocrocin) OR TITLE-ABS-KEY (crocetin) OR TITLE-ABS-KEY (safranal) OR TITLE-ABS-KEY (saffron) OR TITLE-ABS-KEY (“Crocus sativus”) OR TITLE-ABS-KEY (crocin))) AND ((TITLE-ABS-KEY (“Renal function”) OR TITLE-ABS-KEY (“Renal function test”) OR TITLE-ABS-KEY (“Kidney function”) OR TITLE-ABS-KEY (“Kidney function test”) OR TITLE-ABS-KEY (“Blood urea nitrogen”) OR TITLE-ABS-KEY (Creatinine) OR TITLE-ABS-KEY (bun) OR TITLE-ABS-KEY (urea)))
Web of Science
Search hits: 100
(“Renal function” (Topic) or “Renal function test” (Topic) or “Kidney function” (Topic) or “Kidney function test” (Topic) or “Blood urea nitrogen” (Topic) or Creatinine (Topic) or bun (Topic) or cr (Topic)) AND ((picrocrocin (Topic) or crocetin (Topic) or safranal (Topic) or saffron (Topic) or Crocus sativus (Topic) or crocin (Topic)))

**Table 2:** Characteristics of included trials.

First author (publication year)	Country	RCT design	Participants	Sample size case/control	Mean age	Mean BMI (kg/m^2^)	Sex	Intervention of experimental group	Intervention of control group	Duration (weeks)	Statistical adjustments
Kianbakht et al., 2011	Iran	Parallel double-blind	Healthy	45/44	21.8	21.5	M	100 mg/day saffron tablets	Placebo	6	—

Mansoori et al., 2011	Iran	Parallel double-blind	MDD	10/10	38.85	—	M/F	30 mg/day saffron Capsule	Placebo	4	—

Mohamadpour et al., 2013	Iran	Parallel double-blind	Healthy	22/22	31.1	24.9	M/F	20 mg/day crocin tablets	Placebo	4	—

Mousavi et al., 2015 (a)	Iran	Parallel double-blind	Schizophrenia	20/21	49.3	—	M	30 mg/day saffron Capsule	Placebo	12	—

Mousavi et al., 2015 (b)	Iran	Parallel double-blind	Schizophrenia	20/21	49.3	—	M	30 mg/day Crocin Capsule	Placebo	12	—

Milajerdi et al., 2017	Iran	Parallel double-blind	T2DM	27/27	54.99	26.07	M/F	30 mg/day saffron Capsule	Placebo	8	Basal values (income, education, marriage, drug, job, sex, family numbers, WC, weight, BMI, age)

Sepahi et al., 2018 (a)	Iran	Parallel double-blind	T2DM T1DM	20/20	55.74	—	M/F	5 mg/day Crocin tablets	Placebo	12	—

Sepahi et al., 2018 (b)	Iran	Parallel double-blind	T2DM T1DM	20/20	56.63	—	M/F	15 mg/day Crocin tablets	Placebo	12	—

Ebrahimi et al., 2019	Iran	Parallel double-blind	T2DM	40/40	54.1	29.9	M/F	100 mg/day saffron powder	Placebo	12	Baseline values (BMI, WC, durationof T2DM, typesof hypoglycemic drugs), physical activity, and usual dietary intake

Moravej Aleal et al., 2019	Iran	Parallel double-blind	T2DM	32/32	52.95	28.15	M/F	30 mg/day saffron capsule	Placebo	12	—

Karimi-Nazari et al., 2019	Iran	Parallel double-blind	Prediabetes	36/39	57.92	29.06	M/F	15 mg/day saffron tablets	Placebo	8	baseline values (sex, WC, weight, BMI, age, physical activity)

T2DM: Type 2 diabetes, MDD: major depressive disorder, T1DM: type 1 diabetes, NAFLD: nonalcoholic fatty liver disease, M: male, F: female, WC: waste circumference, BMI: body mass index, BUN: blood urea nitrogen, Cr: creatinine.

**Table 3:** Risk of bias assessment for included randomized controlled clinical trials.

First author (publication year)	Random sequence generation	Allocation concealment	Blinding of participants and personnel	Blinding of outcome assessment	Incomplete outcome data	Selective reporting	Other sources of bias	Overall quality
Kianbakht et al., 2011	L	U	L	U	L	L	U	Poor

Mansoori et al., 2011	L	L	L	L	U	L	L	Good

Mohamadpour et al., 2013	U	U	L	L	L	U	L	Poor

Mousavi et al., 2015	L	U	U	U	L	L	U	Poor

Milajerdi et al., 2017	L	L	L	L	L	L	L	Good

Sepahi et al., 2018	L	L	L	L	L	H	L	Fair

Ebrahimi et al., 2019	L	U	L	L	H	L	U	Poor

Moravej Aleali et al., 2019	L	L	L	L	L	L	U	Good

Karimi-Nazari et al., 2019	L	L	L	L	L	L	L	Good

L: low risk, H: high risk, U: unclear.

**Table 4:** GRADE summary of findings.

Outcome	Study design (number of participants)	Risk of bias	Inconsistency	Indirectness	Imprecision (ES (95%CI))	Publication bias	Other	Quality of evidence
Creatinine	11 RCTs (*n* = 588)	Most information is from studies at low or unclear risk of bias	*I* ^2^ = 90.6%, *P* < 0.001	One point	0.04 (−0.01 to 0.09)	Begg's test: *P*=0.639, Egger's test: *P*=0.867	No	Very low

Blood urea nitrogen	10 RCTs (*n* = 499)	Most information is from studies at low or unclear risk of bias	*I* ^2^ = 49.6%, *P*=0.037	One point	−0.69 (−1.36 to −0.01)	Begg's test: *P*=0.655, Egger's test: *P*=0.543	No	Low

GRADE working group grades of evidence. High quality: We are very confident that the true effect lies close to that of the estimate of the effect. Moderate quality: We are moderately confident in the effect estimate: The true effect is likely to be close to the estimate of the effect, but there is a possibility that it is substantially different. Low quality: Our confidence in the effect estimate is limited: The true effect may be substantially different from the estimate of the effect. Very low quality: We have very little confidence in the effect estimate: The true effect is likely to be substantially different from the estimate of effect.

## Data Availability

The data that support the findings of this study are available from the corresponding author upon reasonable request.
